# Structure and evolution of the bifidobacterial carbohydrate metabolism proteins and enzymes

**DOI:** 10.1042/BST20200163

**Published:** 2021-03-05

**Authors:** Shinya Fushinobu, Maher Abou Hachem

**Affiliations:** 1 Department of Biotechnology, The University of Tokyo, Tokyo 113-8657, Japan; 2 Collaborative Research Institute for Innovative Microbiology, The University of Tokyo, Tokyo 113-8657, Japan; 3 Department of Biotechnology and Biomedicine, Technical University of Denmark, 2800 Lyngby, Denmark

**Keywords:** ABC transport proteins, bifidobacterium, carbohydrate metabolism, glycoside hydrolase, host–microbe interactions, human gut microbes

## Abstract

Bifidobacteria have attracted significant attention because they provide health-promoting effects in the human gut. In this review, we present a current overview of the three-dimensional structures of bifidobacterial proteins involved in carbohydrate uptake, degradation, and metabolism. As predominant early colonizers of the infant's gut, distinct bifidobacterial species are equipped with a panel of transporters and enzymes specific for human milk oligosaccharides (HMOs). Interestingly, *Bifidobacterium bifidum* and *Bifidobacterium longum* possess lacto-*N*-biosidases with unrelated structural folds to release the disaccharide lacto-*N*-biose from HMOs, suggesting the convergent evolution of this activity from different ancestral proteins. The crystal structures of enzymes that confer the degradation of glycans from the mucin glycoprotein layer provide a structural basis for the utilization of this sustainable nutrient in the gastrointestinal tract. The utilization of several plant dietary oligosaccharides has been studied in detail, and the prime importance of oligosaccharide-specific ATP-binding cassette (ABC) transporters in glycan utilisations by bifidobacteria has been revealed. The structural elements underpinning the high selectivity and roles of ABC transporter binding proteins in establishing competitive growth on preferred oligosaccharides are discussed. Distinct ABC transporters are conserved across several bifidobacterial species, e.g. those targeting arabinoxylooligosaccharide and α-1,6-galactosides/glucosides. Less prevalent transporters, e.g. targeting β-mannooligosaccharides, may contribute to the metabolic specialisation within *Bifidobacterium*. Some bifidobacterial species have established symbiotic relationships with humans. Structural studies of carbohydrate-utilizing systems in *Bifidobacterium* have revealed the interesting history of molecular coevolution with the host, as highlighted by the early selection of bifidobacteria by mucin and breast milk glycans.

## Introduction

The human gut microbiota (HGM) is a key determinant of the host's health [1–3]. Members of the *Bifidobacterium* genus have attracted significant attention as health-promoting ‘probiotic’ HGM members [4]. Bifidobacteria colonise the guts of social insects (e.g. honey bees) and mammals [5–7], which is supported by co-evolutionary adaptation to these niches, notably, the human gut [8]. Bifidobacteria are saccharolytic, relying on the metabolism of dietary and/or host-derived carbohydrates. Together with other key glycan degraders e.g. Bacteroidaceae, bifidobacteria have maintained symbiosis with their hosts during hominid evolution over 15 million years [9]. The central role of glycan catabolism in this successful adaptation [10,11] is consistent with the abundance of *Bacteroides* and *Bifidobacterium* [12]. Polysaccharide utilization loci (PULs) that encode TonB-dependent oligosaccharide transporters, transcriptional regulators and one or several outer-membrane attached and periplasmic carbohydrate-active enzymes (CAZymes) that target a specific glycan have been proposed to promote human colonisation by *Bacteroides* [13,14]. TonB transporters consist of an integral membrane pore (SusC) and extracellular lipid-anchored glycan-binding protein (SusD) that confers oligosaccharide capture and internalization via SusC [15]. Similarly, genes for carbohydrate utilization and ATP-binding cassette (ABC) transporters are often colocalised in bifidobacteria [16]. ABC transporters consist of dimeric transmembrane domains that form the translocation pore, two cytoplasmic nucleotide-binding domains that energise the uptake, and an extracellular MalE-type substrate-binding protein (SBP, or solute-binding protein) for high specificity and affinity ligand-capture [17]. This review summarizes the current knowledge and recent advances in the bifidobacterial carbohydrate uptake and metabolism apparatus with a focus on structural studies. The evolutionary origins of this protein machinery are also surmised based on structural comparisons of homologs.

## Catabolism of digestible carbohydrates

**Figure 1.**
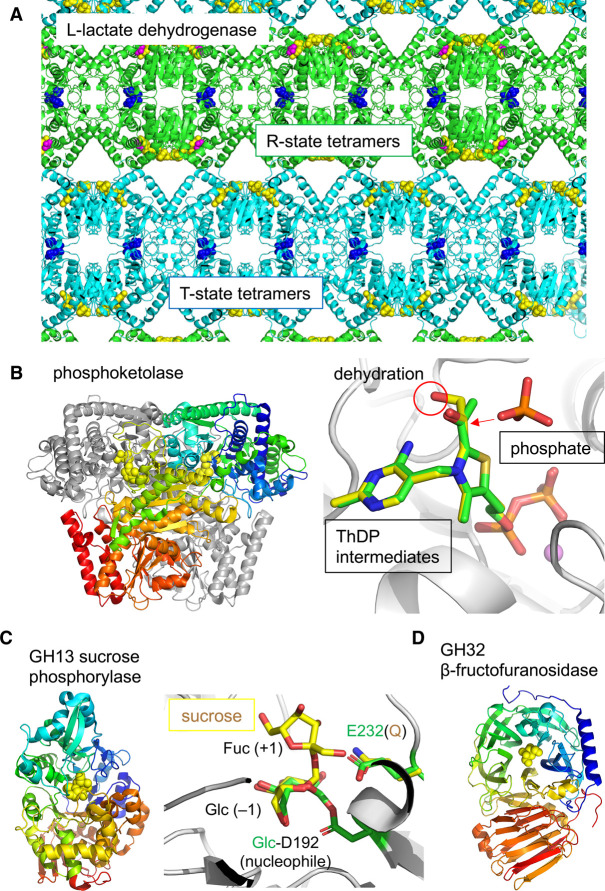
Enzymes for fermentation and digestible carbohydrates. (**A**) Allosteric l-lactate dehydrogenase from *B. longum* aM101-2 (PDB: 1LTH). R-state (green, high substrate affinity) and T-state (cyan, no substrate affinity) tetramers are packed in a single crystal. NADH, FBP, and oxamate (substrate analogue) are shown as yellow, blue, and magenta spheres, respectively. (**B**) Phosphoketolase from *B. breve* 203. Left panel, the overall dimer structure (PDB: 3AHC) is shown with thiamine diphosphate (ThDP) as yellow spheres. Right panel, the active site is shown as a composite of α,β-dihydroxyethyl ThDP (yellow, PDB: 3AHD) and 2-acetyl-ThDP (green, PDB: 3AHE) intermediates and phosphate (PDB: 3AHF). (**C**) GH13 sucrose phosphorylase from *B. adolescentis* DSM20083. Left panel, overall structure (PDB: 2GDU) is shown with sucrose as yellow spheres. Right panel, the active site is shown as a superimposition of a sucrose complex (yellow, PDB: 2GDU) and a covalent glucosyl-enzyme intermediate (green, PDB: 2GDV). The catalytic acid/base residue (Glu232) was mutated to glutamine to obtain the complex with sucrose. (**D**) The overall structure of GH32 β-fructofuranosidase from *B. longum* KN29.1 is shown with β-fructofuranose as yellow spheres (PDB: 3PIJ).

The allosteric l-lactate dehydrogenase, which catalyses the last step of glycolysis, from *Bifidobacterium longum* aM101-2 was the first to be studied from this genus [18]. The structure of 1 : 1 complex of R-state (relaxed, high substrate affinity) and T-state (tense, no substrate affinity) tetramers from a single crystal was reported (Figure 1A) [19,20]. Binding of the allosteric activator, fructose 1,6-bisphosphate, triggers a quaternary structural change of the tetramer, and the substrate affinity is controlled by helix sliding between subunits. Interestingly, bifidobacteria adopt a unique fermentation pathway called the ‘bifid’ shunt [21]. The key bifid shunt enzyme is phosphoketolase, which catalyses phosphorolytic cleavage of fructose 6-phosphate or xylulose 5-phosphate to aldose phosphate, acetyl phosphate, and H_2_O. The crystal structures of phosphoketolases from *B. longum* JCM 1217 and *Bifidobacterium breve* 203 were determined [22–24] (Figure 1B). Phosphoketolase is dependent on thiamine diphosphate coenzyme and catalyses dehydration and phosphorylation reactions. The reaction intermediates were captured by crystal structures, providing the structural basis for this unique catalysis [24].

The first structure of a bifidobacterial glycoside hydrolase (GH) assigned in the Carbohydrate-Active enZymes (CAZy) database [25] was the GH13 sucrose phosphorylase from *Bifidobacterium adolescentis* DSM20083 (Figure 1C) [26]. The reaction mechanism of this enzyme, involving the covalent enzyme-glucosyl intermediate formation and nucleophilic attack by phosphate, was proposed [27]. Concerning the metabolism of fructose-containing carbohydrates, the crystal structure of the GH32 β-fructofuranosidase from *B. longum* KN29.1, which has a typical GH32-type catalytic domain with a five-bladed β-propeller fold, was reported (Figure 1D) [28]. This enzyme releases fructose residues from sucrose, 1-kestose, nystose, inulin, and raffinose *in vitro*. Inulin-type fructans are abundant storage polysaccharides and common dietary fibres. The putative intracellular localisation of the β-fructofuranosidase suggests that inulin oligomers are the likely physiologically relevant substrates of this enzyme.

## Import and degradation of host-derived glycans

### Common metabolic pathway of HMOs and mucin glycans in infant gut-associated bifidobacteria

**Figure 2.**
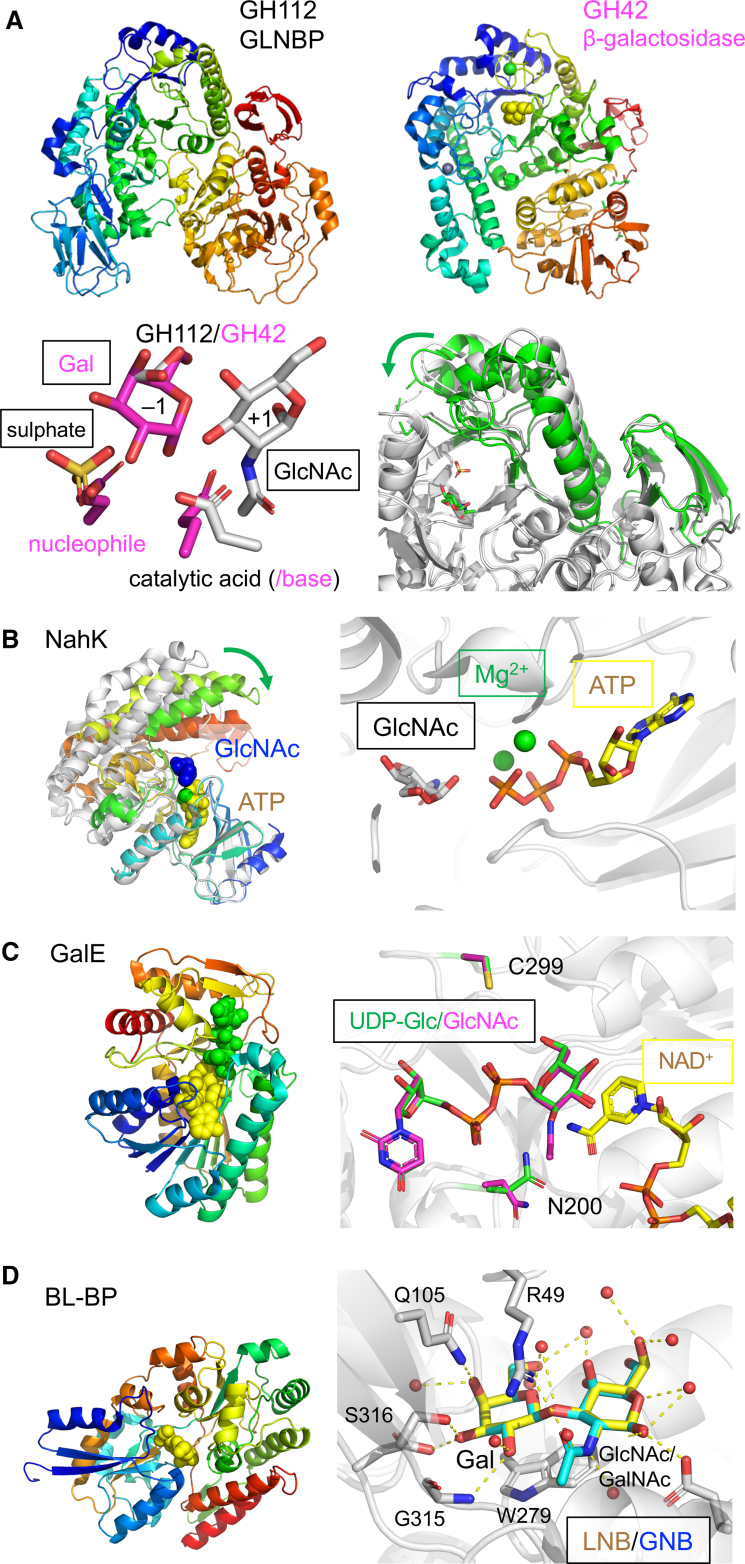
Enzymes and the transporter solute binding protein in the LNB/GNB pathway of *B. longum* JCM 1217. (**A**) The GH112 GLNBP (upper left, PDB: 2ZUS) and the GH42 β-galactosidase (upper right, PDB: 1KWK) with galactose shown as yellow spheres. Bottom left panel, superimposition of the active sites of the GH112 GLNBP (white, composite of PDB: 2ZUV and 2ZUW) and the GH42 β-galactosidase (magenta, PDB: 1KWK). Bottom right panel, the closing motion of the (β/α)_8_ barrel of GLNBP is shown as a superimposition of open (white, PDB: 2ZUS) and closed (green, PDB: 2ZUV) states. (**B**) *N*-acetylhexosamine 1-kinase NahK. Left panel, the closing motion of NahK is shown with open (white, PDB: 4WH3) and closed (rainbow colour, PDB: 4OCJ) states. ATP, Mg^2+^, and GlcNAc are shown as yellow, green and blue spheres, respectively. Right panel, the active site is shown as a composite of GlcNAc (white sticks) and ATP (yellow sticks) plus Mg^2+^ ions (green spheres). (**C**) UDP-glucose 4-epimerase GalE. Left panel, overall structure (PDB: 6K0H) is shown with NAD^+^ and UDP-Glc as yellow and green spheres, respectively. Right panel, the active site is shown as a superimposition of the complex structures with UDP-Glc (green) and UDP-GlcNAc (magenta). NAD^+^ is shown as yellow sticks. (**D**) The transporter GNB/LNB-binding protein (GL-BP). Left panel, overall structure (PDB: 2Z8D) is shown with LNB as yellow spheres. Right panel, the substrate-binding site is shown as a superimposition of the complex structures of LNB (yellow, PDB: 2Z8D) and GNB (cyan, PDB: 2Z8E).

Several *Bifidobacterium* species, e.g. *B. longum* subsp. *longum* (*B. longum*), *B. longum* subsp. *infantis* (*B. infantis*), *B. breve*, and *Bifidobacterium bifidum* are prevalent in infant's guts [6]. *Bifidobacterium*, which is the most abundant genus of the infant gut microbiota, is associated with beneficial effects on infant health [29]. The symbiotic relationship between humans and infant gut-associated bifidobacteria is mediated through mother's milk oligosaccharides (human milk oligosaccharides, HMOs) [30]. HMOs are complex oligosaccharides composed of galactose (Gal), glucose (Glc), *N*-acetylglucosamine (GlcNAc), l-fucose (Fuc), and sialic acid (Neu5Ac) [31]. Lacto-*N*-biose I (Galβ1-3GlcNAc, LNB) is an abundant HMO building block and one of the key units of the symbiosis. The phosphorylase gene that catalyses the cleavage of LNB into α-galactose 1-phosphate and GlcNAc was initially isolated from *B. longum* JCM 1217, and this enzyme became the founding member of the GH112 family [32]. Because this enzyme is also highly active on galacto-*N*-biose (Galβ1-3GalNAc, GNB), it was termed GNB/LNB phosphorylase (GLNBP). GNB is a major core disaccharide unit of *O*-glycoproteins, which are present in human milk fat globules and mucins in gastrointestinal mucus (described below) [33]. The GH112 GLNBP gene is ubiquitous in infant gut-associated bifidobacteria that can grow on LNB [29,34]. The three-dimensional structure determination of GLNBP provided a hint for a possible evolutionary origin of this enzyme [35]. The overall structure and domain architecture of GLNBP are similar to those of GH42 β-galactosidases that are widely distributed in infant gut-associated bifidobacteria (Figure 2A) [36]. Interestingly, the catalytic components of GLNBP at the centre of the catalytic domain are superimposed with those of GH42 β-galactosidases: The phosphate-binding site and the catalytic acid (proton donor) of GLNBP (inverting phosphorylase) are positioned near the nucleophile and the acid/base residues of GH42 β-galactosidase (retaining hydrolase), respectively. Therefore, a possible evolutionary relationship between these galactoside-cleaving enzymes was suggested [35]. The catalytic domain of GLNBP was suggested to undergo a closure motion upon binding of phosphate, which involved a large and unusual deformation of the (β/α)_8_ barrel fold. Such an intricate adaptation to the substrate by a deformation of the (β/α)_8_ fold instead of variations in the loops is a unique case of molecular evolution.

Along with GLNBP, the gene cluster for LNB/GNB metabolism is composed of an ABC transporter and three intracellular enzymes that convert the cleavage products of GLNBP into glycolysis precursors: *N*-acetylhexosamine 1-kinase (NahK), UDP-glucose 4-epimerase (GalE), and UDP-glucose–hexose-1-phosphate uridylyltransferase (GalT) [37]. NahK phosphorylates GlcNAc or GalNAc while galactokinase in the Leloir pathway (a common galactose metabolism route) is specific for galactose [37]. Remarkably, the crystal structure of NahK was not structurally similar to galactokinase that also phosphorylates the sugars at the anomeric C1 position [38]. Note that the first report of NahK structure wrongly designates its name as ‘*N*-acetylhexosamine 1-phosphate kinase’. A large open-close conformational change in NahK was observed, and two Mg^2+^ in the ATP-binding site facilitate the catalysis (Figure 2B) [39,40]. GalE catalyses the interconversion of *gluco*- and *galacto*-hexoses (C4 epimerization) linked to UDP. Crystal structures of GalE from *B. longum* were determined in a complex with UDP-Glc and UDP-GlcNAc (Figure 2C) [41]. GalE has broad substrate specificity due to the large pocket formed by Cys299 and the swing-in/out motion of Asn200. Interestingly, GalE from *B. longum* exhibits functional and structural similarities to the corresponding enzyme from the human host [42]. The bifidobacterial GalT belongs to a minor class of the GalT family (class II) [43], which remains structurally uncharacterized. The SBP of the ABC transporter in the LNB/GNB pathway was also characterized in detail [44]. Since this SBP was highly specific for LNB and GNB, it was termed the GNB/LNB-binding protein (GL-BP). GL-BP has a similar fold with the maltose binding protein (SBP fold), and both LNB as well as GNB are extensively recognized by the protein (Figure 2D) [44].

### Divergent strategies of HMO utilization

**Figure 3.**
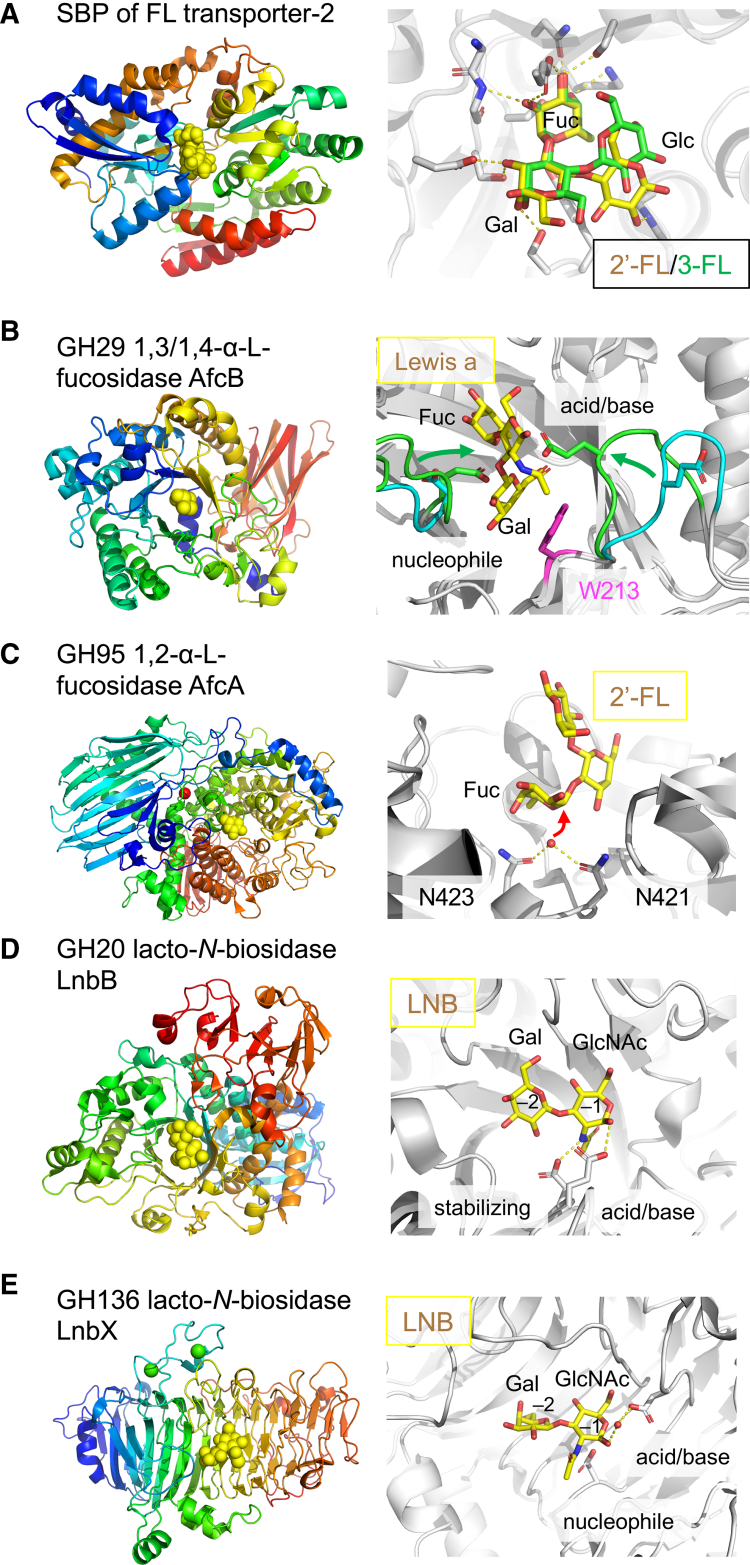
Enzymes and SBP for HMO utilization by infant gut-associated bifidobacteria. (**A**) Fucosyllactose binding protein (SBP) of the FL transporter-2 from *B. infantis* JCM 1222. Left panel, overall structure (PDB: 6HUR) shown with 2′-fucosyllactose (2′-FL) as yellow spheres. Right panel, the substrate-binding site is shown as a superimposition of the complex structures with 2′-fucosyllactose (2′-FL, yellow, PDB: 6HUR) and 3-fucosyllactose (3-FL, green, PDB: 6HUS). (**B**) GH29 1,3/1,4-α-L-fucosidase AfcB from *B. infantis* ATCC 15697. Left panel, overall structure (PDB: 3UET) is shown with fucose as yellow spheres. Right panel, the induced-fit motion of the active site loops shown as a superimposition of apo (cyan, PDB: 3MO4) and complex (green, PDB: 3UET) structures with Lewis a trisaccharide (yellow sticks). The side chains of the catalytic residues (green sticks) were modelled from the D172A/E217A double mutant structure. The aromatic stacking platform for Gal (Trp213, magenta) is also shown. (**C**) GH95 1,2-α-L-fucosidase AfcA from *B. bifidum* JCM 1254. Left panel, overall structure (PDB: 2EAC) is shown with deoxyfuconojirimycin as yellow spheres. Right panel, the active site is shown as a composite of the 2′-FL complex (PDB: 2EAD) and the wild-type enzyme (PDB: 2EAC). 2′-FL, two catalytic asparagine residues, and the nucleophilic water are shown as yellow sticks, white sticks, and a red sphere, respectively. (**D**) GH20 lacto-*N*-biosidase LnbB from *B. bifidum* JCM 1254. Left panel, overall structure (PDB: 4H04) is shown with LNB as yellow spheres. Right side, the active site showing LNB. (**E**) GH136 lacto-*N*-biosidase LnbX from *B. longum* JCM 1217. Left panel, overall structure (PDB: 5GQF) is shown with LNB as yellow spheres. Right side, the active site showing LNB. The side chains of the catalytic residues are shown in (**D**) and (**E**).

Bifidobacteria, which generally possess fewer extracellular enzymes than *Bacteroides*, rely on ABC transporters to import dietary oligosaccharides. The infant gut-associated *B. infantis* and *B. breve* use ABC transporters for uptake and subsequent intracellular digestion of HMOs [29,45]. *B. infantis* JCM 1222 has two ABC transporters for uptake of the major HMOs, fucosyllactose (FL). The prevalent FL transporter-2 has a broader specificity than the less common counterpart [46]. The crystal structures of the FL transporter-2 SBP complexed with 2’-FL and 3-FL revealed the recognition of a disaccharide Fuc-[Gal/Glc] motif, with the Fuc unit residing at the bottom of the binding cleft (Figure 3A). These findings rationalise the basis for the dual transporter specificity.

The intracellular GH29 1,3/1,4-α-L-fucosidase in *B. infantis* ATCC15697 (AfcB) effectively cleaves the 3-fucosyl and 4-fucosyl substitutions of HMOs [47]. The crystal structure of AfcB revealed a prominent galactose-binding site that contributes to selective binding of the Lewis a and x epitopes in HMOs or other host glycans (Figure 3B) [48]. AfcB exhibits unique induced-fit loop motions to position the catalytic nucleophile and acid/base residues [48].

Compared with the two infant gut-associated species described above, *B. longum* and *B. bifidum* adopt an ‘extracellular digestion’ strategy [49]. In particular, *B. bifidum* strains behave as altruistic members in the gut microbiota ecosystem by sharing HMO degradants produced by their extracellular glycosidases [50,51]. For example, the GH95 1,2-α-l-fucosidase from *B. bifidum* JCM 1254 (AfcA) specifically cleaves the α-1,2-linked fucosyl residues of HMOs or related host glycans [52]. The overall structure of AfcA (anomer-inverting GH, Figure 3C) exhibited a striking resemblance to inverting glycoside phosphorylases of GH65 and GH94 [53]. The active site of AfcA has a unique feature involving two asparagine residues that were suggested to activate the water nucleophile [53]. For the degradation of LNB-containing (type 1) HMOs, *B. bifidum* has a key extracellular enzyme called lacto-*N*-biosidase (LnbB), which belongs to GH20 and hydrolyses the central β1,3 bond of lacto-*N*-tetraose to produce LNB and lactose [54]. The structure of LnbB (Figure 3D) differ from canonical monosaccharide-releasing GH20 exo-β-*N*-acetylhexosaminidases [55] by having an extra –2 subsite that allows the accommodation of the nonreducing galactosyl in LNB. The *N*-acetyl group of GlcNAc adopted a distorted conformation to be poised for nucleophilic attack on the anomeric C1 atom, consistent with the substrate-assisted mechanism in GH20.

*B. longum* has a special adaptation among infant gut-associated bacteria because it is also widely distributed in the adult and elderly human gut [56]. The recently discovered extracellular lacto-*N*-biosidase from *B. longum* JCM 1217 (LnbX) is not homologous to GH20 LnbB and thus represents the founding member of GH136 [57]. LnbX adopts a β-helix fold (Figure 3E) and a retaining mechanism [58]. The active site pocket architecture of LnbX is distinct from other polymer-acting β-helix endo-glycosidases, which have a long cleft. Although the evolutionary origin of LnbX is enigmatic, the protein fold has significant structural similarity to tailspike proteins from bacteriophages, which occasionally possess carbohydrate-binding ability [58]. GH136 LnbX is inactive unless it is coexpressed with an adjacent gene. The homologous lacto-*N*-biosidase from *Eubacterium ramulus* indicated that GH136 enzymes are likely to occur either as heterodimers or as two-domain enzymes, with the second domain/subunit adopting a previously unknown helical fold and contributing to the active site architecture [59].

### Mucin degradation

**Figure 4.**
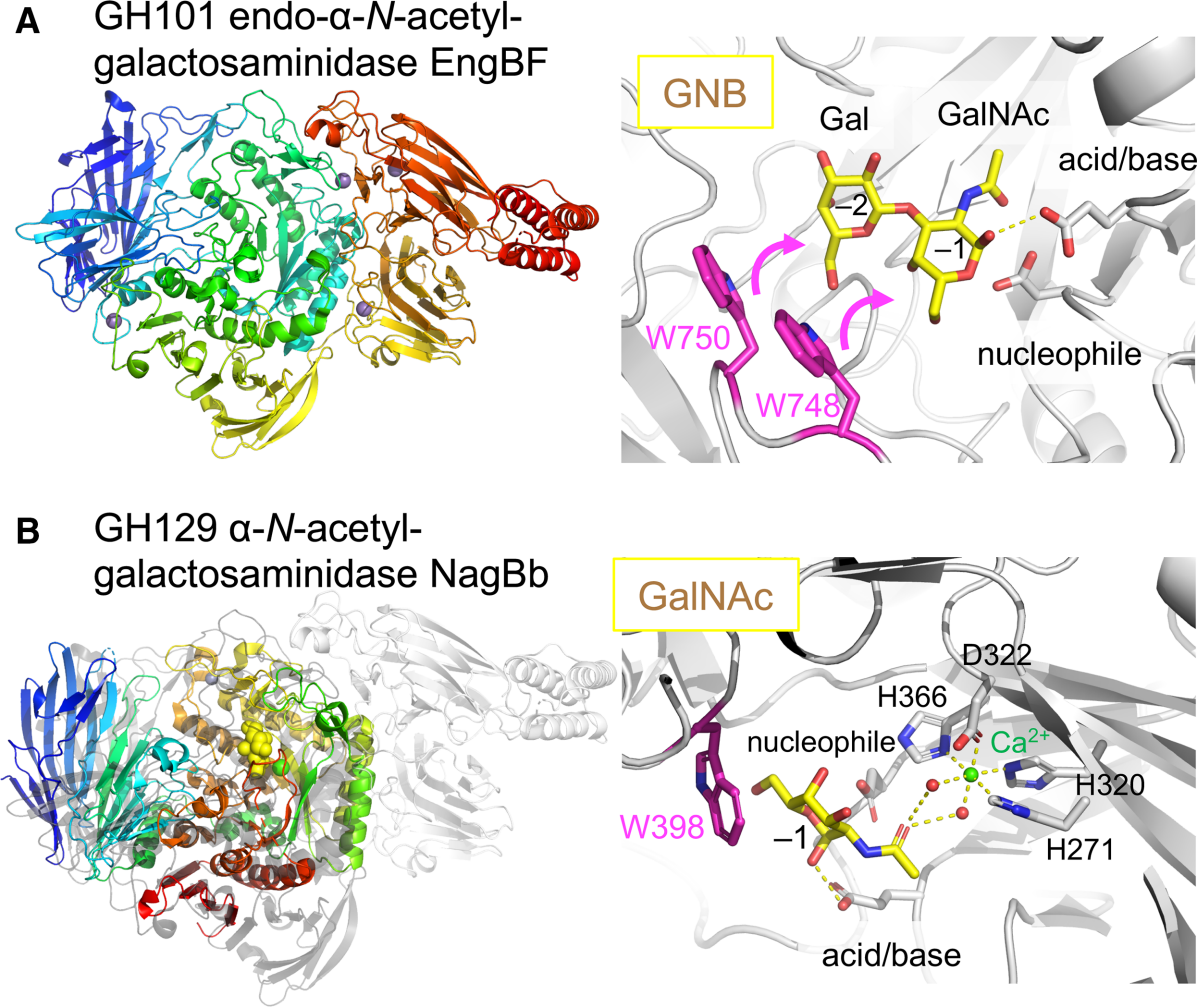
Enzymes for mucin degradation. (**A**) GH101 endo-α-*N*-acetylgalactosaminidase EngBF from *B. longum* JCM 1217. Left panel, overall structure (PDB: 2ZXQ). Right panel, the active site showing GNB modelled by automated docking (yellow sticks). The two tryptophan residues (magenta) form aromatic stacking interactions with the disaccharide. (**B**) GH129 α-*N*-acetylgalactosaminidase NagBb from *B. bifidum* JCM 1254. Left panel, overall structure (PDB: 5WZN) is shown with GalNAc and Ca^2+^ as yellow and green spheres. GH101 EngBF (grey) is overlayed to show the structural similarity. Right panel, the active site showing GalNAc (yellow sticks), Ca^2+^ (green sphere), waters, and protein ligands of Ca^2+^. The aromatic stacking platform for GalNAc (Trp398, magenta) is also shown. The side chains of the catalytic residues are also shown in (**A**) and (**B**).

The mucus layer barrier that protects the epithelium serves also as a sustainable nutrient supply for distinct bacteria via its mucin glycoproteins [60]. Long-term coevolution was likely established due to a mutually beneficial relationship between the human host and the mucin-adherent microbiota [61]. Strains of *B. longum*, *B. bifidum*, and *B. breve* possess glycosidases targeting *O*-linked mucin glycans [62,63]. The three-dimensional structures of two bifidobacterial mucin-degrading enzymes have been reported. The extracellular GH101 endo-α-*N*-acetylgalactosaminidase from *B. longum* JCM 1217 (EngBF) releases the α-linked GNB disaccharide from the Ser or Thr residues in the core 1 units (or T-antigen) [64], which allows for GNB catabolism through the LNB/GNB pathway. The catalytic domain of EngBF has a partially broken (β/α)_8_ barrel similar to GH13 α-amylases (Figure 4A) [65]. Two active site tryptophans stack onto GNB by an induced-fit motion [66]. The second mucin-degrading enzyme structure was determined for the GH129 α-*N*-acetylgalactosaminidase from *B. bifidum* JCM 1254 (NagBb) [67], which releases the α-linked GalNAc from the Tn antigen [68]. The catalytic domains of NagBb and GH101 enzymes are similar (Figure 4B). Recognition of the GalNAc is supported by Ca^2+^ and stacking-platform of a tryptophan.

### Import and degradation of plant glycans

Exposure to complex glycans from vegetables and fruits triggers a pronounced expansion of the infant HGM diversity during weaning [69]. The abundance of bifidobacteria decreases after weaning, but plant dietary glycan utilizers remain relatively abundant in adults. Distinct bifidobacteria, e.g. *Bifidobacterium animalis* subsp. *lactis* (*B. lactis*) and *B. longum* subsp. *longum,* are commonly used commercial probiotics. This section focuses on the bifidobacterial proteins that target abundant dietary plant saccharides.

### Common pathways for the utilization of plant dietary saccharides

**Figure 5.**
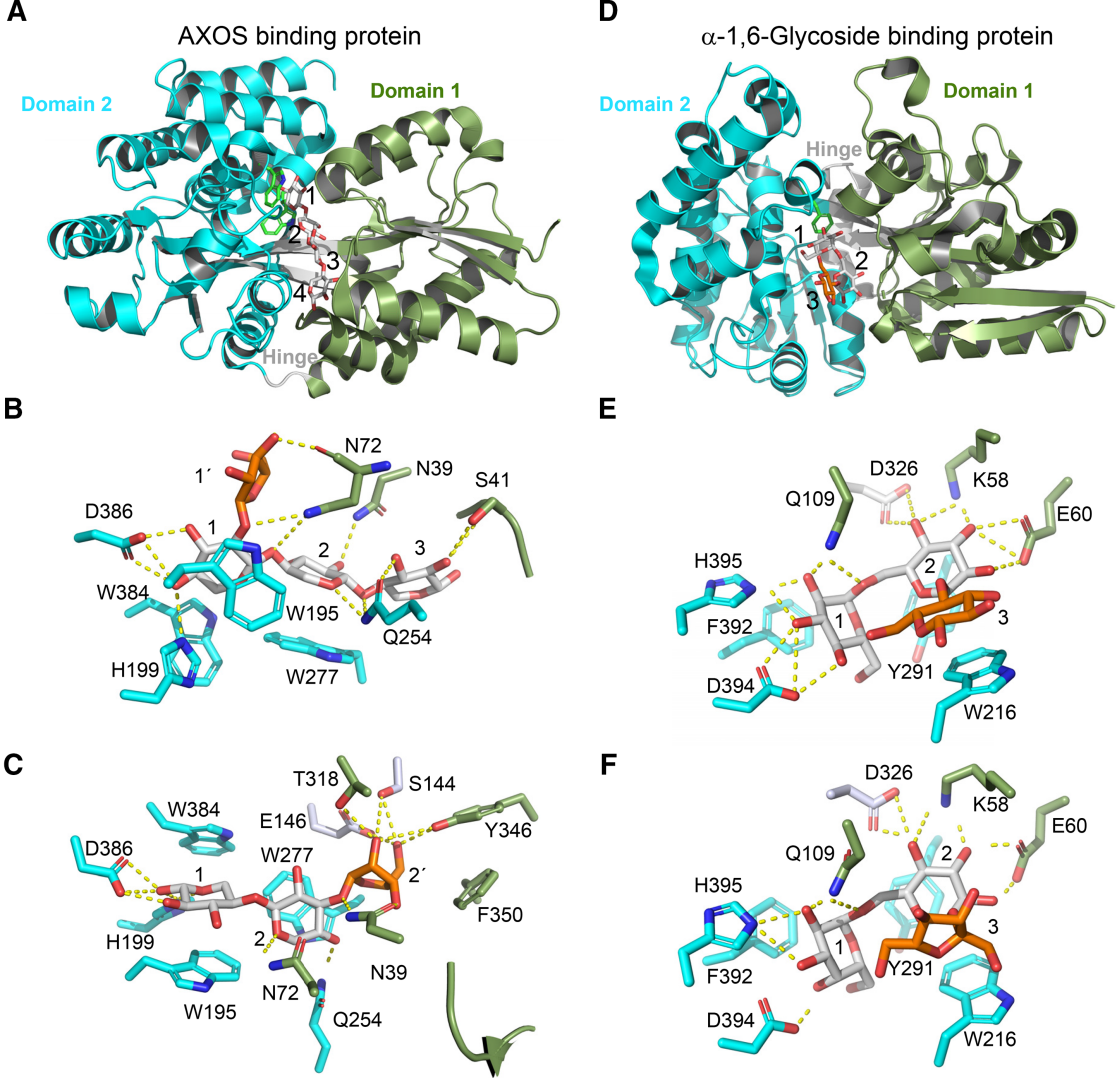
SBPs for abundant plant-derived oligosaccharides by *B. animalis* subsp. *lactis* Bl-04. (**A**) The overall structure of the arabinoxylooligosaccharide binding protein *Bl*AXBP in complex with xylotetraose (PDB: 3ZKK). (**B**) and (**C**) Recognition of arabinoxylotriose (PDB: 4C1T) and arabinoxylobiose (PDB: 4CIU), respectively, with the amino acid residues coloured according to the domain colour as in (**A**). The arabinosyl sidechains (orange) can be accommodated in cavities in the binding protein, and the structure view in (**C**) is tilted approximately 90° for clarity. (**D**) The overall structure of the SBP *Bl*G16BP that mediates the capture of both α-1,6-linked galactosides and glucosides in bound form with the preferred ligand panose (PDB: 4ZZE). (**E**,**F**) Details of binding panose and raffinose (PDB: 4ZS9), respectively. The recognition motif of *Bl*G16BP is the disaccharide unit at positions 1 and 2, and the open binding site allows the accommodation of different monosaccharide units at position 3 (Glc and Fru at position 3 are shown in orange).

Arabinoxylan is a major dietary fibre from cereal cell walls. Only a few primary degraders from *Bacteroides* [70], *Roseburia intestinalis* [71] and *Eubacterium* can degrade xylan. Bifidobacteria have a conserved genetic locus that confers growth on arabinoxylan oligomers (arabinoxylooligosaccharides, AXOS). This locus is common in the *B. longum*, *B. adolescentis,* and *Bifidobacterium pseudolongum* (that includes *B. lactis*) groups [72]. The AXOS locus, which encodes an ABC transporter, esterases, arabinofuranosidases, xylosidases and xylose catabolism enzymes, was shown to be transcriptionally upregulated in *B. lactis* grown on XOS [73]. The binding protein (*Bl*AXBP) of this transporter has a preference for a xylotetraose (*K*_d_ ≈ 100 nM) and terminal nonreducing end arabinosyl substitutions [72]. Both arabinoxylobiose and arabinoxylotriose were bound with *K*_d_ ≈ 100 nM. The crystal structures of *Bl*AXBP in complex with xylotriose, xylotetraose, 3^2^-α-l-arabinofuranosyl-xylobiose, and 2^3^-α-l-arabinofuranosyl-xylotriose assume a canonical SBP fold (cluster B-I [74]) comprising two domains connected with a tripartite hinge region. The binding site of *Bl*AXBP accommodates a xylotetraose backbone and a flexible lid-loop adjusts its conformation to bind different ligands (Figure 5A). The most densely recognized xylosyl is sandwiched between two tryptophans and recognized by Asp386 acid and His199, which define subsite 1 that is occupied in all structures (Figure 5B,C). Cavities flanking subsites 1 or 2 allow binding of C2 or C3-arabinosyl substituents. The AXOS backbone can be accommodated in two opposite directionalities, which expands the range of captured AXOS. Altogether, these features contribute to the versatility of AXOS capture.

Raffinose family oligosaccharides from soybean and isomaltooligosaccharides from starch degradation, honey, or dextran breakdown are abundant in the human gut. Both oligosaccharide classes share a [Gal/Glc]α1,6Glc motif and promote the growth of bifidobacteria, except for *B. bifidum* [75]. The most transcriptionally upregulated locus during the growth of *B. lactis* [73] on both α1,6-glycosides is conserved in other bifidobacteria except *B. bifidum*, consistent with the observed growth profiles [75]. This locus encodes a transcriptional regulator, an α-galactosidase [76], a GH13_31 α-1,6-glucosidase [77], and an ABC transporter. Uptake of oligosaccharides sharing a [Gal/Glc]α1,6Glc via the ABC transporter [75] was corroborated by the crystal structures of the associated α1,6-glycoside binding protein (*Bl*G16BP) in complex with raffinose (Galα1,6Glcα1,2βFru) and panose (Glcα1,6Glcα1,4Glc) (Figure 5D–F). Compared to AXOS binding in a cleft, the binding site of *Bl*G16BP features a deep pocket [75]. Asp394 confers the dual recognition of galactosyl or glucosyl units at subsite 1 via hydrogen bonds to either the axial or equatorial C4-OH, respectively (Figure 5E,F). The C-terminal domain harbours an aromatic stacking platform and Asp394 and His395 that recognise the saccharide unit at position 1. The recognition of the glucosyl at subsite 2 in both panose and raffinose is identical, whereas the lack of direct polar bonds at subsite 3 is consistent with the plasticity at this site. *B. lactis* outcompeted *Bacteroides ovatus* after 18 h of growth on raffinose in a 50 : 50 starting mixed culture [75], which underscores the role of ABC transporters in conferring competitive growth on preferred substrates.

### Specialization of distinct bifidobacteria on certain plant glycans

**Figure 6.**
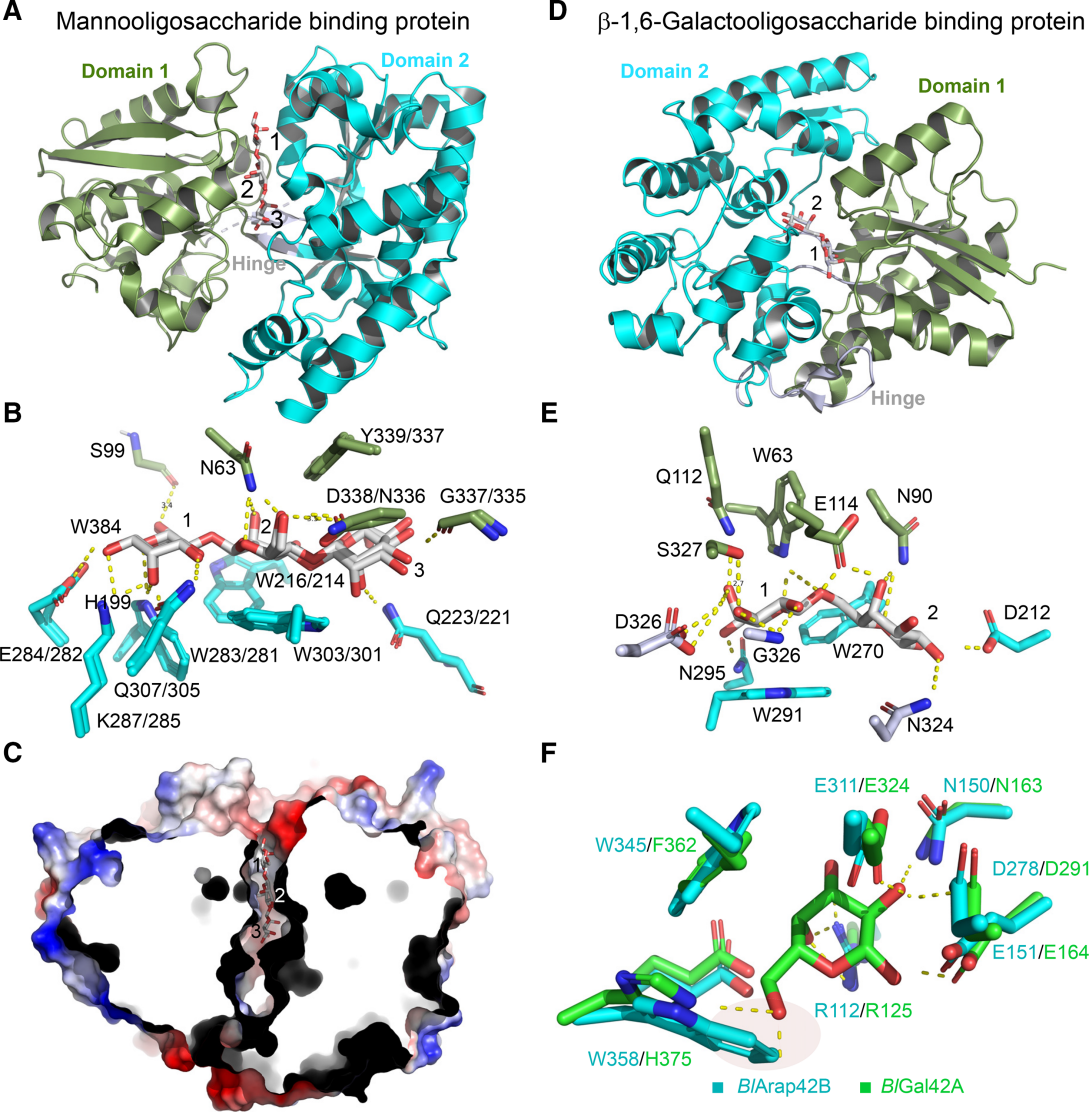
Transporter SBPs and enzymes of specialized utilization for plant-derived glycans by *B. animalis* subsp. *lactis*. (**A**) Structure of the mannooligosaccharide binding protein from *B. animalis* subsp. *lactis* ATCC 27673 (*Bl*MnBP1) in complex with mannotriose (PDB: 6I5V). (**B**) Differences in the binding site of the two homologous binding proteins *Bl*MnBP1 and *Bl*MnBP2 (PDB: 6FUV) that display high and low binding affinity to mannobiose, respectively. The amino acid residues are coloured according to the domain colour in (**A**). *Bl*MnBP1/*Bl*MnBP2 amino acid identities and numbering are shown. Asn63 is present only in *Bl*MnBP1 and interacts with the mannosyl at subsite 2. (**C**) Cross-sectional view of the electrostatic surface of *Bl*MnBP1 cocrystallised with mannopentaose (PDB: 6I5W) reveals additional space and cavities beyond subsite +3, consistent with the binding profile of larger or galactosyl-decorated oligosaccharides. (**D**) and (**E**) show the overall structure of the binding protein *Bal*6GBP (PDB: 6H0H) in complex with β1,6-galactoside and the detailed recognition of this ligand, respectively. (**F**) Comparison of the active site subsite –1 of the recently discovered α-l-arabinopyranosidase *Bl*Arap42B (PDB: 5XB7) and the β-1,6/1,3-β-galactosidase *Bl*Gal42A (PDB: 4UNI) in complex with galactose. A key difference is the substitution of a histidine that recognizes the C6-OH with a hydrogen bond in *Bl*Gal42A with a tryptophan residue that makes a steric clash with the C6-OH and instead is likely to stack onto the arabinopyranose that lacks this group.

β-Mannans are structural and storage plant polysaccharides, which are heavily used as food additives. Primary degraders of mannan include *Bacteroides ovatus* [78] and *R. intestinalis* [79]. Mannan utilisation is not as widespread in *Bifidobacterium* as arabinoxylan or α1,6-glycosides discussed above. A predicted cell-attached GH26 mannanase from *B. adolescentis* [80] and a secreted GH5 mannanase from *B. lactis* have been biochemically characterized [81]. The growth of *B. lactis* ATCC 27673 on konjac glucomannan and galactomannan from carob bean gum has been reported [82]. Proteomic analysis revealed the upregulation of a GH5 mannanase, a putative β-mannosidase, a β-glucosidase, as well as an ABC transporter with two homologous SBPs during growth on mannan [82]. The affinity of one of these binding proteins (*Bl*MnBP1) to mannobiose was 2500-fold higher compared to the second (*Bl*MnBP2). Otherwise, both proteins had similar binding profiles with a preference for a mannotriose and binding of galactomannooligosaccharides with a galactosyl at the reducing mannosyl unit (*K*_d_ ≈ 2 μM), which is consistent with the growth on galactomannan. The structures of *Bl*MnBP1 and *Bl*MnBP2 in complex with mannobiose/mannotriose and mannotriose, respectively [82], revealed similar binding sites (Figure 6A,B). The mannosyl specificity is mediated by hydrogen bonds to the axial C2-OH at subsites 1 and 2, whereas a spacious cavity beyond subsite 3 accommodates galactosyl sidechains (Figure 6C). The most striking difference is the substitution of an asparagine (Asn63) that is hydrogen-bonded to the mannosyl at subsite 2 in *Bl*MnBP1 to glycine in *Bl*MnBP2 (Figure 6B). Mutational analyses supported the role of this residue in substrate binding. This study highlighted the differentiation of SBP specificities via duplication and one or several mutations that alter the affinity/specificity to distinct substrates.

The role of oligosaccharide transporters has been also investigated in the specialization of *B. lactis* Bl-04 towards β1,6-galactooligosaccharides. The preferred β1,6-galactobiose conferred rapid and immediate growth of *B. lactis* Bl-04, while the less preferred β1,3 and β1,4 isomers resulted in several hours-long lag phases. The galactooligosaccharide locus in this strain comprises an ABC transporter and a GH42 β-galactosidase (*Bl*GH42A) [73], which was shown to prefer β1,6-galactobiose, thus defining a new specificity in GH42 [83]. This enzyme had comparable catalytic efficiencies (within 5-fold) towards the β1,3- and the β1,4-galactobiose compared to the preferred β1,6-isomer. Galactose binding at subsite -1 induced a conformational change in the loop region Asn202-Gln206, allowing the formation of a hydrogen bond to the C4-OH of the bound galactose.

In contrast to the enzymatic promiscuity of *Bl*GH42A, the ABC transporter's SBP (*Bal*6GBP) is strikingly selective towards β1,6-galactobiose (*K*_d_ = 92 nM), with 300- and 1630-fold lower affinities towards the β1,3- and β1,4-linked isomers, respectively [84]. The structure of *Bal*6GBP was determined in complex with β1,6-galactobiose (Figure 6D). The β1,6-galactobiose ligand is recognized by 12 potential hydrogen bonds, with all the OH-groups of the nonreducing galactosyl (subsite 1) being engaged in hydrogen bonds (Figure 6E). Interestingly, galactosyl binding at subsite 1 is conserved in *Bal*6GBP and the *B. longum* LNB binding protein (GL-BP) (Figures 2D and 6E). The substitution of Trp63 in *Bal*6GBP with Arg49 in GL-BP from *B. longum* correlates with the change in specificity at position 2 from Gal in *Bal*6GBP to GlcNAc in GL-BP.

The catabolism of plant-derived bioactive glycosides by the HGM exerts an important impact on human health. The mechanisms of this metabolic facet remain largely unexplored except in a few studies e.g. in *Lactobacillus acidophilus* [85]. Recently, a new GH42 α-l-arabinopyranosidase from *B. lactis* (*Bl*Arap42B) was discovered [86]. *Bl*Arap42B releases α-l-arabinopyranoside from bioactive plant glycosides, e.g. paenolide or ginsenoside Rb2. This specificity reflects the structural similarity between α-l-arabinopyranoside and β-d-galactopyranoside. The only difference is the loss of C6-OH in the former. Indeed, the space occupied by the C6-OH in β-galactosidases is blocked with the indole side chain of Trp358 in *Bl*Arap42B (Figure 6F). These active site differences revealed the signatures underpinning the diversification of specificities with GH42.

### β-Arabinooligosaccharides

**Figure 7.**
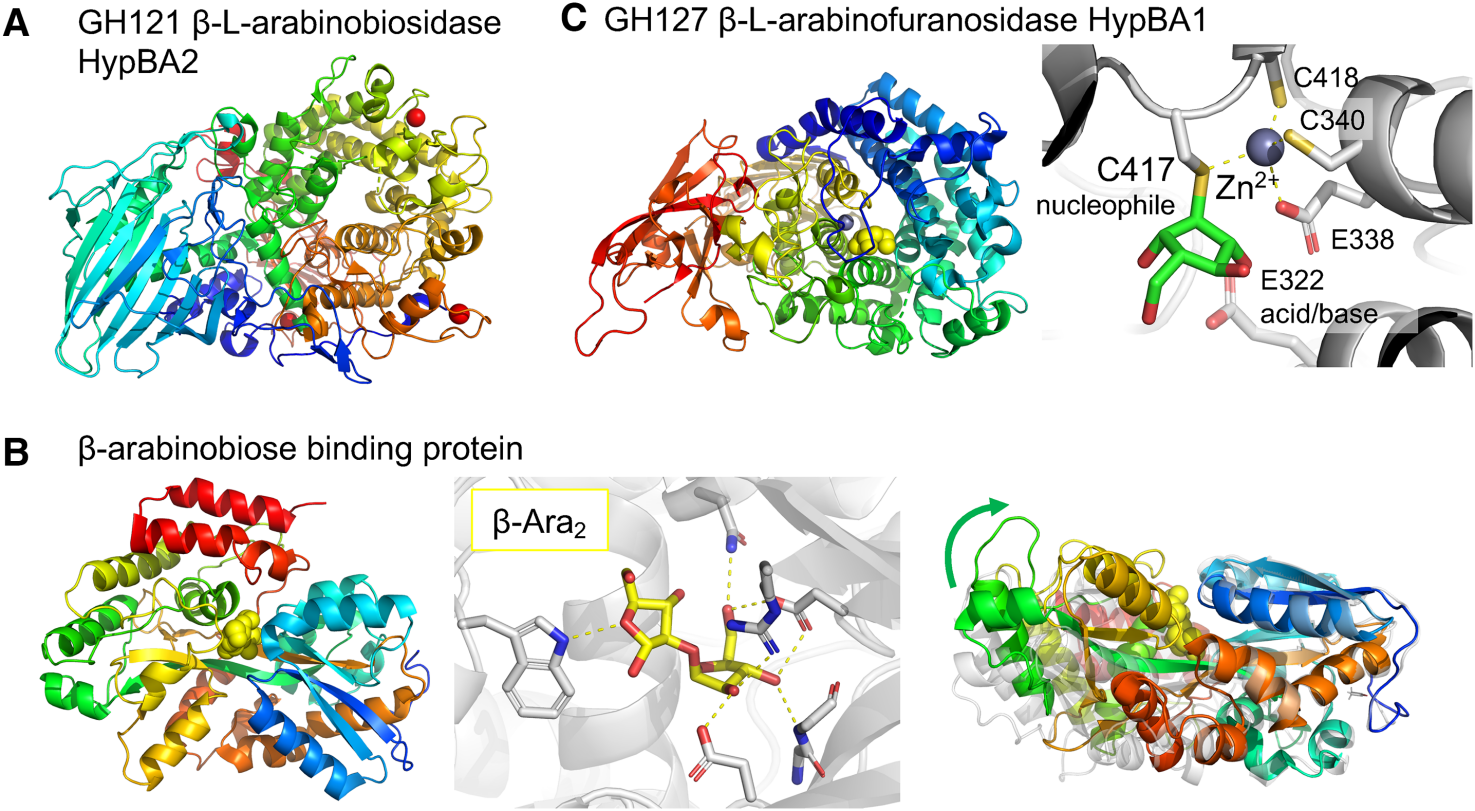
Enzymes and SBP for β-arabinooligosaccharides utilization by *B. longum* JCM 1217. (**A**) Overall structure of GH121 β-l-arabinobiosidase HypBA2 (PDB: 6M5A). (**B**) β-Arabinobiose binding protein. Left panel, overall structure (PDB: 6LCE) showing β-Ara_2_ as yellow spheres. Middle panel, the substrate-binding site showing β-Ara_2_ (yellow sticks). Right panel, closing motion is shown by the superimposition of the liganded structure (rainbow colour) with the open ligand-free state (white) calculated by a molecular dynamics simulation. (**C**) GH127 β-l-arabinofuranosidase HypBA1 (PDB: 3WKX). Left panel, overall structure showing β-l-arabinofuranose as yellow spheres. Right panel, the active site structure. The covalent intermediate structure with a cyclophellitol-derived inhibitor (PDB: 7DIF) is shown. Cys417 and Glu322 are the catalytic nucleophile and the acid/base residues.

The diverse human diet promotes the evolution of unique pathways in bifidobacteria to specialise on less common plant-derived carbohydrates. *B. longum* JCM 1217 possesses a gene cluster encoding the utilisation of β-linked arabinofuranooligosaccharides [87]. β-Arabinoligosaccharides are present in plant cell wall components called hydroxyproline-rich glycoproteins, including expansin and lectins from edible plants. The utilisation system comprises an extracellular enzyme that releases β-1,2-linked arabinofuranose disaccharide (β-Ara_2_; GH121 β-l-arabinobiosidase HypBA2), which is internalised by an ABC transporter and an intracellular enzyme that cleaves the disaccharide into monomers (GH127 β-l-arabinofuranosidase HypBA1) [88]. The catalytic domain of GH121 HypBA2 adopts an (α/α)_6_ barrel fold (Figure 7A), similar to several GH families [89]. Three acidic (glutamate and aspartate) residues in the active site pocket are essential for catalysis. The SBP of the ABC transporter (β-arabinobiose-binding protein) exclusively binds β-Ara_2_ via extensive interactions, and its dynamic feature was investigated by computational simulation (Figure 7B) [90]. The crystal structure of the retaining GH127 HypBA1 revealed an unprecedented ‘cysteine glycoside hydrolase’, which uses a cysteine residue as the catalytic nucleophile (Figure 7C) [91]. Although its catalytic domain has a typical (α/α)_6_ barrel fold, the catalytic site has a unique architecture composed of a Zn^2+^ coordinated by (Cys)3-Glu. Cys417 is closely positioned to the anomeric C1 of β-l-arabinofuranose and identified as the possible nucleophile. A detailed mechanistic study using a cyclophellitol-derived inhibitor revealed the covalent glycosyl-enzyme intermediate structure and the itinerary of the catalytic reaction of HypBA1 [92].

## Conclusions

The crystal structures of other bifidobacterial glycosidases were also reported: GH3 β-glucosidase from *B. longum* KACC 91563 for saponin metabolism [93], GH5 β-mannosidase from *B. longum* NCC2705 for *N*-glycan utilization [94], and GH42 β1,6-galactosidase [95] and GH43 exo-β1,3-galactanase from *B. bifidum* S17 [96] for degradation of β-galactoside-containing carbohydrates in the human diet. Here we presented examples of the structural studies performed to date on carbohydrate-related enzymes and transporter binding proteins from the *Bifidobacterium* genus. However, considering their surprisingly broad carbohydrate utilisation capabilities, our structural and biochemical insights into these catabolic pathways remain limited; thus, further investigation of such enzymes and transport proteins is required. Bifidobacteria have been highly successful in establishing symbiotic relationships with different hosts, most notably humans. Therefore, deciphering the structure-based mechanisms of the protein components is instrumental for revealing the interesting molecular coevolution history between bifidobacteria and their hosts.

## Perspectives

- *Importance to the field*: A considerable expansion of pathways targeting host-derived or dietary plant glycans is observed in *Bifidobacterium*. Although bifidobacteria are considered secondary degraders with few predicted extracellular glycosidases, they are equipped with an arsenal of ABC transporters that are crucial for competitive growth on diverse oligosaccharides.
- *Summary of current thinking*: Although some of the transporters of bifidobacteria have been broadly shared within the genus, others have evolved to drive specialization within specific groups. The ability of substrate-binding proteins to capture ligands with high affinity and specificity is likely to be important for efficient cross-feeding.
- *Future directions*: Bifidobacteria have been widely used as commercial probiotics, but the molecular mechanisms responsible for their symbiosis with humans and associated health benefits remain underexplored. Insight into the species- and/or strain-specific metabolic signatures will be instrumental for designing personalized mechanism-based dietary interventions to target distinct bifidobacteria and promote human health.

## Open Access Statement

Open access for this article was enabled by the participation of the University of Tokyo in an all-inclusive *Read & Publish* pilot with Portland Press and the Biochemical Society under a transformative agreement with USACO.

## Abbreviations

ABC: ATP-binding cassette
AXOS: arabinoxylooligosaccharides
CAZymes: carbohydrate-active enzymes
FL: fucosyllactose
GH: glycoside hydrolase
GNB: galacto-*N*-biose
HGM: human gut microbiota
HMOs: human milk oligosaccharides
LNB: lacto-*N*-biose I
SBP: substrate- or solute-binding protein
